# Ethical and Legal Considerations in the Collection and Analysis of Mental Health Lived Experience Narratives: Reflections on 4 Case Studies of Research Practice

**DOI:** 10.2196/89452

**Published:** 2026-09-16

**Authors:** Stefan Rennick-Egglestone, Laura E R Blackie, Laura Asher, Rahel Birhane, Wubalem Fekadu, Donna Franklin, Ian James Kidd, Mark Pearson, Elvira Perez Vallejos, Mike Slade

**Affiliations:** 1 Institute of Mental Health, School of Health Sciences, University of Nottingham, Triumph Road, Nottingham, England, United Kingdom, 44 7504331005; 2 National Institute for Health and Care Research (NIHR) Nottingham Biomedical Research Centre, Nottingham, England, United Kingdom; 3 School of Psychology, University of Nottingham, Nottingham, England, United Kingdom; 4 Academic Unit of Lifespan and Population Health, School of Medicine, University of Nottingham, Nottingham, England, United Kingdom; 5 WHO Collaborating Centre for Mental Health Research & Capacity Building, Department of Psychiatry, School of Medicine, College of Health Sciences, Addis Ababa University, Addis Ababa, Ethiopia; 6 Centre for Innovative Drug Development and Therapeutic Trials for Africa (CDT-Africa), Addis Ababa University, Addis Ababa, Ethiopia; 7 Sheffield Centre for Health and Related Research (SCHARR), Division of Population Health, School of Medicine, University of Sheffield, Sheffield, England, United Kingdom; 8 Department of Philosophy, University of Nottingham, Nottingham, England, United Kingdom; 9 Lincolnshire Unit for Mental Health Research (LUMHR), University of Lincoln, Lincoln, England, United Kingdom; 10 School of Medicine, University of Nottingham, Nottingham, England, United Kingdom; 11 School of Computer Science, University of Nottingham, Nottingham, England, United Kingdom; 12 Health and Community Participation Division, Faculty of Nursing and Health Sciences, Nord University, Namsos, Nordland, Norway

**Keywords:** lived experience, mental health, narratives, narrative inquiry, narrative intervention, responsible innovation, research ethics, legal

## Abstract

Personal narratives describing lived experiences of the entire spectrum of mental health challenges are now widely available to the public, including through autobiographies from public figures, thematic collections of narratives assembled by mental health organizations, and individual narratives published on video sharing services. Mental health lived experience narratives have been used as an “active ingredient” in interventions intended to create change, such as in campaigns against mental health stigma. In the narrative inquiry research approach, they are used to explore mental health phenomenology. Researchers and organizations working with mental health lived experience narratives have to contend with a wide range of legal and ethical challenges, such as how to handle narratives disclosing sensitive personal information about third parties and the ethical trade-off between preserving narrator autonomy over their presentation of personal identity and protecting narrators from harm due to mental health stigma if a narrator is identifiable in their narrative. In 2022, we formed the Interdisciplinary Consortium on Narratives in Context (ICONIC) of people with knowledge of narrative practices across disciplines. Members are engaged in health research, liberal arts, modern languages, history, and philosophy. In this viewpoint, we present four case studies of narrative practices by ICONIC members: (1) a narrative inquiry into the experiences of Ethiopian citizens with schizophrenia, (2) work to curate and share 2 collections of mental health recovery narratives, (3) an exploration of the use of poetic transcription to condense narrative interviews with mental health content, and (4) the development of safe approaches to working with personal narratives shared through an online mental health peer support service. In presenting these case studies, we focused on documenting decision-making on ethical and legal challenges as the best knowledge on ethical and legal decision-making regarding mental health lived experience narratives may come from integrating knowledge across disciplines. Through reflecting on these case studies, we identified cross-cutting challenges regarding consent processes, narrative analysis, and the interpretation and dissemination of data and findings. These challenges have transdisciplinary and disciplinary-specific features and can be used as a preliminary checklist in research design processes. In presenting what we learned, our intent was to demonstrate that greater knowledge on narrative practices can emerge through interdisciplinary contact and inform future decision-making on narrative practices by researchers working across disciplines. We conclude by contemplating interdisciplinary explorations with the potential to expand knowledge, including examining the use of pathographic narratives in philosophical inquiry.

## Introduction

Personal narratives describing lived experiences of the entire spectrum of mental health challenges are now widely available to the public. Examples include autobiographies from public figures [1,2], thematic collections of narratives assembled by mental health organizations and activists [3,4], and individual narratives published on video sharing services [5-7]. Changes due to influential narratives have been documented, including increased self-advocacy for a bipolar diagnosis due to commonalities in experience with those described in an autobiography [8]. An enhanced capacity for self-advocacy through exposure to the experiences of others may be contributing to increased diagnosis rates for neurodevelopmental conditions [9]. There is a debate on whether this increased recognition reflects unmet need or contributes to harm through overdiagnosis [10].

Media reception theory views people as playing an active role in decoding (interpreting and making meaning from) media they have received [11]. For mental health lived experience narratives, the Narrative Experiences Online (NEON) impact model describes some specific mechanisms and outcomes through which decoding takes place and creates personal change [12]. Validation of the NEON impact model was conducted through a randomized controlled trial of access to an online mental health narrative collection that found significant increases in quality of life and meaning in life and also reductions in psychological distress for female participants [13]. Because lived experience narratives can create change, they have been used as an active ingredient [14] in interventions. A systematic review identified 5 domains of intended change in health care and community settings (political, societal, community, service level, and individual) [15]. A pedagogical review documented uses including illustrating complex concepts or supporting personal connection to course content [16].

Many of the published uses of lived experience narratives with the intention of creating change have been led by organizations seeking to create mental health benefit. Some organizations creating narrative collections have been heavily resourced. For example, Time to Change [17], a UK antistigma campaign integrating online lived experience narratives, received at least £20 million in public funding (£1=US $1.36 as of August 26, 2026). The potential influence of well-resourced organizations on how mental health issues are seen and understood makes their decision-making worthy of study. Our previous systematic review [18] and interview study [19,20] documented categories of decision represented by the acronym VOICES (values and motivations, organization, inclusion and exclusion, control and collaboration, ethics and legal, and safety and well-being). Through our own work to curate a collection of mental health narratives [12], we have found that decisions in the VOICES “ethics and legal” category require the most sustained attention.

When working with mental health narratives, legal issues are prominent as these narratives frequently contain personal information categorized as sensitive in current legal frameworks such as the General Data Protection Regulation (GDPR) [21] and can reveal sensitive information about third parties. Ethical issues include decisions about the representation of narrator identity, where curators may need to balance imperatives such as respecting narrator autonomy with avoiding predictable harms due to disclosure [18], particularly when web-based technologies allow for the rapid dissemination of sensitive content. Narratives are frequently used in research to understand health phenomenology through an approach known as narrative inquiry [22]. Here, curatorial decisions can influence the nature of knowledge produced and, hence, raise ethical concerns. For example, a previous systematic review on the characteristics of mental health recovery narratives included 45 documents. All but 2 were either analyses of narratives collected through interviews or published as prose [23], meaning that the research community risks omitting mental health knowledge from people less able to verbalize in the moment or produce written text [24].

In 2022, we formed the Interdisciplinary Consortium on Narratives in Context (ICONIC) to bring together people with knowledge relevant to an understanding of narrative practices, including researchers and members of the public [25]. ICONIC was interdisciplinary by design, spanning research disciplines with an interest in narrative, including (but not limited to) interventional and observational health research, liberal arts, modern languages, history, and philosophy. Through our meetings, we reflected on case studies of our own narrative practices, including to identify decisions made and approaches taken. We found that issues of ethics and legality were frequently a focus of consortium members in their practices, particularly for those issues for which there was no clear best practice or the potential for harm.

The aim of this paper is to describe and discuss some of the ethical and legal issues that we have identified through the work of ICONIC, illustrated through 4 case studies of the work of consortium members selected for relevance to these challenges. Our intent is to demonstrate that greater knowledge on narrative practices can emerge through interdisciplinary contact and inform future decision-making about narrative practices by researchers working across disciplines.

## Case Studies of Narrative Practice

### Case Study 1: Narratives Describing Schizophrenia Recovery in Ethiopia

The Rehabilitation Intervention for People With Schizophrenia in Ethiopia (RISE) cluster randomized controlled trial evaluated the effectiveness of a 12-month community-based rehabilitation program in improving disability among people with severe mental illness in rural Ethiopia [26-28]. To explore experiences of personal recovery in this setting, RISE conducted 52 narrative interviews with 13 linked pairs of individuals with severe mental illness and their primary caregivers at baseline and 12 months [29]. People with severe mental illness were asked to share their experiences over time, describing preillness, help seeking, and treatment; hopes for the future; and barriers to and enablers of desired changes. Caregivers were usually family members who held a primary role providing care due to the lack of mental health services across the region [30,31]. They were asked to comparatively describe their perceptions of their relatives’ experiences. This case study, therefore, amounts to a narrative inquiry into the experiences of a specific group of people, where the work of the people conducting the narrative inquiry was informed by their knowledge of the specific needs of participants.

### Case Study 2: Curation of 2 Collections of Mental Health Recovery Narratives

The NEON study curated a collection of 659 narratives describing personal recovery from mental health problems for use within research that examined whether access to the narratives in this collection could help people affected by mental health problems. This included 3 clinical trials that evaluated whether web-based access to NEON collection narratives benefited people affected by mental health problems [13,32,33]. NEON collection narratives were donated by individuals and sourced from existing published collections such as books and websites. Almost all the narratives in the NEON collection were already in the public domain. To curate this collection, the NEON study team developed consent procedures, inclusion criteria, and decision-making processes [12]. The NEON team also collected and analyzed 80 recovery narratives from groups underrepresented in research [34]. For both datasets, the NEON team developed approved procedures for sharing narratives with other researchers. These included documents specifying practices required of those researchers, such as the inclusion of people with personal experiences of mental health challenges in analytical processes. All the development described above took place through internal discussion and iterative engagement with people with experience curating narrative collections; people with lived experience of mental health problems; and the study sponsor, who governed the ethical and legal conduct of the study. The portion of the work of NEON presented in this case study, therefore, amounts to an action research exploration of narrative practices that integrated the perspectives of a range of experts.

### Case Study 3: Poetic Transcription of Mental Health Narratives

As a creative and reflexive approach to data interpretation in a PhD thesis [35], a researcher created poems from narrative interview transcripts that had previously been considered through Labovian narrative analysis [36]. This work built on a tradition of poetic transcription, which has been described as “the creation of poem-like compositions from the words of interviews” [37] and has also been termed “poetic condensation” [38]. Poetic transcriptions have been generated from narratives to engage and evoke the imaginations of audiences [39] and provide unique insights into the personal experiences of research participants [40]. An example from the PhD thesis is provided in Multimedia Appendix 1. Through the work described in this case study, the researcher carefully reflected on the ethical and legal issues inherent in producing new creative material from existing narratives.

### Case Study 4: Recommendations for Responsible Research Use of Online Platforms Integrating Peer Support Narratives

There are a range of online platforms that enable people experiencing mental health problems to receive support from peers with similar experiences. Discussions on these platforms frequently involve the sharing of personal narratives of mental health problems, frequently in the form of shorter or longer narratives. While such platforms can offer a rich environment for researchers to test out their research questions and hypotheses, the perceived privacy, intimacy, and sensitivity of the content that they hold urges researchers and platforms to uphold the highest ethical standards as they undertake responsible research practices [41] that go beyond the legal requirements within platforms’ terms and conditions. A case study examined how to work responsibly with the Kooth online platform [42] and proposed a series of recommendations [43].

## Ethical and Legal Considerations Identified in the Case Studies

### Overview

Table 1 summarizes the ethical and legal considerations that we identified across the 4 case studies. These have been grouped into 3 categories reflecting different phases of the research process: consent procedures, narrative analysis and interpretation, and dissemination of data and findings.

In synthesizing these 4 case studies, we did not attend to considerations on data curation as these have been addressed in depth in our systematic review on narrative uses and misuses [15].

**Table 1.** Ethical and legal considerations present in the 4 case studies.

	Case study 1: RISE^a^ trial	Case study 2: NEON^b^ study	Case study 3: poetic transcription	Case study 4: online platforms
Consent procedures				
Transparency and understanding of consent procedures	✓			✓
Tensions between legality of consent and privacy expectations		✓		✓
Legality of receiving and maintaining consent for narrative curation		✓		✓
Narrative analysis and interpretation				
Importance of social context for interpretation	✓			✓
Coconstruction or cocreation of meaning	✓		✓	
Dissemination of data and findings				
Data ownership		✓	✓	
Authenticity of social reality and experience			✓	
Anonymization of data to permit sharing		✓		✓

a RISE: Rehabilitation Intervention for People With Schizophrenia in Ethiopia.

b NEON: Narrative Experiences Online.

### Considerations Regarding Consent Procedures

Obtaining appropriate consent for the collection of narratives raised challenges. In case study 1 (the RISE trial), narratives were collected directly by researchers for research purposes through procedures that involved the provision of participant information and the collection of informed consent. Although obtaining informed consent is an ethical standard in health research, its significance is perhaps amplified within interview-based narrative research given that interviews elicit detailed information as data about participant experiences that may feel deeply connected to personal identity. There may be a particularly high risk of harm to participants if individuals do not fully understand the implications of a study or how the data collected and conclusions drawn through their narratives will be disseminated. This was amplified for case study 1, which was conducted in a setting (rural Ethiopia) where 40% of the population is not formally educated and, hence, lacks literacy, with consent procedures predominately reliant on verbal explanation by research staff or relatives with some formal education [44]. In this setting, people with severe mental illnesses are often generally disempowered by a lack of autonomy and involvement in decision-making processes about their care or life [45,46], meaning that any problems with research consent procedures risked exacerbating previous inequities. Hence, the researchers invested substantial effort in maximizing the clarity of their consent procedures and the materials they used.

Case studies 2 (NEON study) and 4 (online platforms) demonstrate tensions between the legality of the consent process and individuals’ own expectations for reasonable privacy. In case study 4, researchers accessed sensitive narratives shared publicly online but where service users had not shared them for research purposes. To enable ethical access, the researchers worked with the online service provider to insert functionality to collect additional consent rather than relying on clauses buried in a complex “terms and conditions” document that indicated that data could be used to “improve their services” and shared with third parties. Even though it may have been legal to access consented data via uninformed procedures, the researcher felt it morally wrong to access these data without expressed and informed consent; it is widely recognized that online users may not fully attend to or understand the nature or scope of the activities to which they have legally consented [47], and in at least one case, public concern has been raised about an analysis conducted on the basis of clauses in a social media site “terms and conditions” statement [48].

In case study 2, both collections consisted of narratives describing recovery from mental health problems, with a variety of approaches to narrator and third-party identifiability. For example, the NEON collection received some donations of narratives that were pseudonymous and others that directly identified the narrator or third parties by name or through included events or descriptions that would enable the easy identification of either. Given the mental health focus of these narratives, this means that narrative donations frequently contained sensitive personal data as defined in the GDPR. An additional complexity was that some donations included sensitive personal information about third parties whom the narrator had never met but who had inspired their recovery. For example, some narratives referenced the British actor Stephen Fry, who has described his mental health experiences in a range of influential autobiographical books [1]. The legality of this issue was further complicated because, ethically, the research team had committed to not modifying a narrative and respecting narrator choice on self-identification. Each of these commitments excluded a choice to remove sensitive personal information from these narratives.

To address these considerations, the research team developed comprehensive consent procedures, inclusion criteria, and decision-making processes for narrative inclusion and processing to use their collection in research. Their eventual choice was to ensure that they had valid narrator consent to process and share sensitive personal information, to exclude narratives presenting sensitive personal information about third parties, but not to exclude narratives where the research team had collected direct evidence that third-party information had been “manifestly made public by the data subject.” The latter is a term included in article 9 of the GDPR [21] (clause 2e), which removes a prohibition on the processing of sensitive personal data, including sensitive personal data related to the health of the data subject. This allowed the research team to include narratives in which the narrator described the beneficial impact on the self of learning about others’ mental health experiences (eg, as described in Stephen Fry’s autobiography) but to exclude narratives where that information had not been made public (eg, a named person that the narrator had encountered on a mental health ward). Furthermore, the research team only shared these narratives with other researchers for the purposes of secondary research analysis in cases in which they had documented consent to do so.

For a smaller collection of NEON narrative interviews that were collected through researcher interviews, the team took a different path to enable sharing with other researchers for secondary analyses. They had previously pseudonymized these data for analysis through a protocol approved before they realized the importance of giving narrators control over their identity. To enable sharing with other researchers, they then increased transcript anonymity by redacting information about events described in the narrative where there was a reasonable possibility that the narrator could be identified through comparing or combining public records of those events with content described in the narrative. This process was approved by the supervising ethics committee and study sponsor and was legally correct as it placed the anonymized transcript outside of data protection regulations. The study investigator was responsible for confirming the appropriateness of redactions, with their decisions auditable by the study sponsor.

### Considerations Regarding Narrative Data Analysis and Interpretation

Case study 1 (the RISE trial) illustrates the ethical importance of situating the analysis and interpretation of narratives within the context in which they were constructed and collected. While this applies to the collection and analysis of qualitative data more broadly, narrative analysis is a person-centered approach that emphasizes the particularity of human experience [49], which requires careful attention to the social-cultural or political context [50] that influences those experiences. Similar concerns were present in case study 4 (online platforms), where narrative exchanges between forum contributors were situated in the unique interactional context of the forum itself, which was governed by a set of rules put in place and enforced by the service provider through its moderation team. Analyses of these narratives, therefore, need to account for this framework of rules.

The imperative of attending to the social context and the coconstruction of meaning in narrative data is further illustrated in case studies 1 (RISE trial) and 3 (poetic transcription). In case study 3, this coconstruction took the form of the creative interpretation of an individual’s experiences through the transcription process of translating narrative interviews into poetry. This process raised unique issues regarding the accuracy of representation in experiences (or truthfulness) and data ownership that are further discussed in the next section. However, case study 1 raised the issue of ensuring that researchers’ narrative analyses gave voice to their participants while acknowledging that knowledge generated through research is coconstructed through a multitude of perspectives. There was an ethical imperative to faithfully represent both the experience of participants and their sensemaking [51]. However, narratives were collected from Ethiopia—a collectivist society in which the focus is on group obligation and interpersonal harmony over and above autonomy and self-expression. In this context, the family, rather than the individual, has been described as the smallest autonomous unit for decision-making [52]. The neglect of collectivist values in understanding recovery in favor of individualistic principles has been highlighted [53].

The research team, therefore, faced a challenge in deciding whether and how to integrate the caregiver data into the analysis, balancing a person-centered approach with the contextual importance of caregivers. It is inherently undermining to seek “corroboration” from a caregiver and suggests that the researchers view their participants as “unreliable narrators.” Researchers should also not assume that details are unintentionally omitted from narratives by participants, but rather that the process of composing a narrative is a creative act of representing experience [54,55]. In this dataset, some sensitive and potentially stigmatizing details of an individual’s life were reported only by the caregiver. What is selected or performed through narratives reveals how an individual wants to be seen and is, in turn, shaped by the master narratives that researchers wish to interrogate. Furthermore, not only does the caregiver not have greater access to a more authentic “truth,” but they are also often distinctly nonneutral with respect to the individual’s story. Finally, overemphasis on caregiver perspectives may lead to skewed insights for future interventions that do not address the needs of people with severe mental illness. Taking together these ethical questions, the researchers decided to conduct the analysis primarily using the data of the person with severe mental illness. The caregivers were conceptualized predominantly as a key “voice” (ie, a character in the story rather than the teller of the story) [26].

### Considerations Regarding the Dissemination of Data and Findings

Finally, we move to discussing the ethical and legal considerations involved in the publication and dissemination of interpretations and the public sharing of narrative curations with the broader scientific community or public. The ethical issue of data ownership pertaining to whom the data belong to—the participant or the research team—is applicable in several of our case studies but is perhaps best illustrated in case study 3 involving the poetic transcription of mental health narratives. In this context, the narrative is transformed into a new artistic or creative expression, such as a poem. The narrative itself is not disseminated; instead, the poem is. Elliott [56] suggests that the researcher enters, at least tacitly, into a moral relationship with participants that is not solely related to data collection but spans the whole process of research. Within this relationship, consideration should be given to questioning to whom the data belong [57] or, in this case, to whom the poem belongs. For example, in the poem shared in case study 3 (through Multimedia Appendix 1), it could be argued that both the researcher and the participant are the authors. These issues further reinforce the importance of the consent procedures and, ideally, engaging with participants about these issues early in the research cycle before the data have been collected.

In scientific disciplines, the context for case studies 1 (RISE trial) and 2 (NEON study), data sharing is becoming commonplace and more of an expected practice even with sensitive qualitative data. While a research team or study sponsor might own and be responsible for the original data they collect, some research funders and journals encourage these data to be transferred into a form where they can be shared with others for the greater scientific good but with suitable adjustments due to their sensitive content. The ethical and legal issues regarding anonymizing narrative data to protect participants’ identities before sharing them with a broader community outside of the research team have some unique challenges, as illustrated in our earlier discussion on the consent procedures for case study 2. The research team or sponsor must decide on appropriate anonymization measures and communicate these clearly to research participants in their consent procedures. While standard practice with qualitative data is to redact personal information to protect the identities of individuals, this poses some unique challenges with certain narratives, where individuals may view this process as a destructive retelling of their story.

When considering the dissemination of research findings and interpretations through research articles and presentations, several of our case studies raised the importance of engaging with ethical questions regarding the portrayal of truthfulness of human experiences and social realities. In case study 3, the ethical issue of truthfulness was represented in the fact that the construction of the poem arguably involved a partial loss of the original narrative. Poetic transcription does not attempt to include all elements of the interview transcript but rather focuses on the critical elements of the narrative [58], with the aim of representing a more embodied sense of the narrative [38]. In refining the data into stanzas, there is the potential to highlight aspects that might have been ignored when encountered in the data’s original composition [59]. Additionally, this process of poetic transcription may provide unique insights into participants’ narratives that might otherwise have been lost if the data had been subjected solely to thematic forms of analysis [60], wherein the search for shared or contrasting experiences across a group of participants may dissociate the narratives from the individuals [61]. However, there is also the potential for this poetic process to distort the original narrative or for aspects of the narrative to become overemphasized as researchers accentuate the transformative, subversive, or unique qualities of the narrative [62]. Furthermore, the readers themselves may become drawn into an affective fallacy where they conflate the content of the poem with their own emotive response [63].

## Conclusions

Taken collectively, the case studies presented in this paper demonstrate some of the legal and ethical issues that researchers need to consider when collecting or sourcing narrative data for their research. As researchers design new studies, the ethical and legal considerations that we documented in Table 1 can be used as a preliminary checklist that draws attention to critical issues to consider. For example, for the analysis phase of a study, Table 1 draws attention to a need to consider how meaning will be cocreated from narratives, and we encourage study teams to plan for and document issues regarding ownership of narratives and narrative-based knowledge before their collection and analysis begins.

We encourage other researchers interested in narrative analysis, narrative inquiry, and interventional uses of narratives to extend this preliminary list of ethical and legal issues to develop a thorough account of how to work ethically and legally with narrative data in all their forms. Finally, we highlight that the preliminary checklist that we outlined in Table 1 was developed considering existing practices from 4 case studies across different academic disciplines. While some ethical and legal issues were shared across disciplines, we firmly believe that the interdisciplinary approach to narrative research methodology applied in this paper identified issues of research ethics and integrity that may not have necessarily been considered by any single discipline.

While we focused on mental health narratives in this paper, narratives are used as a source and intervention across a wide range of health experiences and research disciplines, and hence, reaching out beyond mental health has the potential to extend knowledge on ethical and legal challenges regarding lived experience narratives. For example, philosophical practice has drawn on pathographic narratives such as Audre Lorde’s autopathography, *The Cancer Journals* [64]. The subtleties of philosophical work on pathographic narratives and the ethical issues with which they must engage, therefore, have the potential to inform narrative practice in health research directly.

## Supplementary material

10.2196/89452 Multimedia Appendix 1 Sample poetic transcription.

## Abbreviations

GDPR: General Data Protection Regulation
ICONIC: Interdisciplinary Consortium on Narratives in Context
NEON: Narrative Experiences Online
RISE: Rehabilitation Intervention for People With Schizophrenia in Ethiopia
VOICES: values and motivations, organization, inclusion and exclusion, control and collaboration, ethics and legal, and safety and well-being
