# Supplementary material for: Ethical and Legal Considerations in the Collection and Analysis of Mental Health Lived Experience Narratives: Reflections on 4 Case Studies of Research Practice
Source: JMIR Form Res. 2026 Sep 16;10:e89452. doi: 10.2196/89452 (PMC13580890; doi:10.2196/89452)
Supplement: Supplementary file 1 [file formative-v10-e89452-s001.docx]

# Sample poetic transcription

The following poetic transcription was created from a narrative interview in which the participant shared experiences of trauma, psychosis, and discovering the value to their recovery of writing their own poetry:

My childhood was very uneven,

Attachment

Abandonment,

That sort of stuff,

My first run in with mental health at 14,

Very psychotic,

Wasn’t letting it out of the bag,

She’s just a young teenage girl seeking attention

First baby at 15

Demons in the baby

I would write little poems

Helped me not to feel scared,

Nearly 17,

I have a toddler,

Locking up the house,

I made it through another night,

I didn’t die,

Collect them,

I read them back to myself,

I survived,

I’m such a survivor

I started to work on the trauma

Little bits of psychosis

I think I’ve got better at managing it,

Abuser,

I’m making him really ugly in poetry,

Fairy tales,

Everything in my favour,

Bringing that pain out in a poem,

See it,

Such

Deep

Trauma,

What the fuck are you doing?

I’m a mess,

Fuck you,

I gotta get better for you,

No one is going to listen,

Need another strategy,

Poetry - people want to read it,

Mother fucker, I’m putting that poem here.

This poem was first published in Pearson [36].
